# Metabolic Abnormalities in a Cohort of Overweight and Obese Children in an Urban Setting of Sri Lanka

**DOI:** 10.1155/2021/9936889

**Published:** 2021-07-05

**Authors:** Loretta S. Warnakulasuriya, Dulani L. Samaranayake, Adikaram V. N. Adikaram, Manel M. A. Fernando, Elisabet Rytter, Iris Ciba, Peter Bergsten, Anders H. Forslund, K. D. Renuka Ruchira Silva, Vithanage Pujitha Wickramasinghe

**Affiliations:** ^1^Postgraduate Institute of Medicine, University of Colombo, Colombo, Sri Lanka; ^2^Department of Community Medicine, University of Colombo, Colombo, Sri Lanka; ^3^Health Unit, Bandaranaike International Airport, Katunayake, Sri Lanka; ^4^Colombo North Teaching Hospital, Ragama, Sri Lanka; ^5^Clinical Nutrition and Metabolism, Department of Public Health and Caring Science, Faculty of Medicine, Uppsala University, Uppsala, Sweden; ^6^Department of Women's and Children's Health, Uppsala University, Uppsala, Sweden; ^7^Uppsala University Children's Hospital, Uppsala, Sweden; ^8^Department of Medical Cell Biology, Uppsala University, Uppsala, Sweden; ^9^Department of Applied Nutrition, Wayamba University of Sri Lanka, Makandura, Gonawila, Sri Lanka; ^10^Department of Paediatrics, Faculty of Medicine, University of Colombo, Colombo, Sri Lanka

## Abstract

Childhood obesity-related metabolic derangements are increasing among South Asian populations. Most of these changes persist to adulthood. This study aims to describe the distribution of metabolic abnormalities among 7- to 17-year-old overweight and obese children in the Gampaha District of Sri Lanka. Overweight children (age- and gender-adapted BMI>+1SD, WHO standards) were selected from a community survey carried out in the Negombo Education Zone of Gampaha District. After a 12-hour overnight fast, blood was drawn, and blood glucose (FBG), lipid profile, insulin, and liver transaminases were measured. Two hours after a glucose load, blood was drawn for random blood glucose (RBG) and insulin. Metabolic syndrome (MetS) was diagnosed using modified IDF criteria for children. Anthropometry, fat mass (FM), and blood pressure were measured. Hepatic fat pattern was assessed ultrasonically. The data of 403 children (210 boys) were analysed. Of the study population, 16.4% were overweight (BMI for age +1 to +2SD), 72% were obese (BMI for age >+2 to +3SD), and 11.6% were severely obese (BMI for age >+3SD). Insulin resistance was seen in 46.8%, and prevalence increased with age. Mean postprandial insulin ranged from 368 to 625 pmol/L and was elevated in 35%. Dysglycaemia was seen among 20.8%. MetS was present in 19.8%, and 84% had at least one metabolic abnormality. Different degrees of hepatic steatosis were observed in 32.5%, and elevated ALT/AST ratio was seen in 58% of the population. Overweight and obesity during childhood were associated with multiple metabolic abnormalities including MetS, and they occur from a young age. It is important to screen children for overweight/obesity early in life and intervene to prevent them from developing metabolic complications.

## 1. Introduction

Obesity, one of the primary risk factors for noncommunicable diseases (NCDs), is spreading in epidemic proportions all over the world, penetrating rapidly into the paediatric population [1]. The prevalence of obesity has doubled in many countries since 1980 [1]. Although the prevalence of childhood obesity is lower than that of adults, the rate of increase is very high among children compared to adults. Between 1980 and 2015, there was a significant relative increase of 20.0% in the prevalence of obesity in countries with a low socio-demographic index and the highest rates of increase were observed in countries with a middle socio-demographic index [2]. Although the national rates of childhood obesity are still low in Sri Lanka, there are certain geographical areas, which show increased prevalence. Data from Colombo municipal area showed overweight and obesity to be about 14% among 8- to 12-year-old school children [3] and in Colombo district about 13% among 5- to 15-year-old children [4]. In the Negombo education zone, overnutrition was shown to vary between 10 and 18% across 5–15 year age group with the prevalence increasing with age [5].

The epidemic of obesity and its related comorbidities have adversely impacted children and adolescents all over the world. Childhood obesity is a major risk factor for the development of adult obesity and many NCDs such as diabetes, hypertension, dyslipidaemia, and some forms of cancer later in life [6]. Total body fat mass (FM) of Sri Lankan children is high compared to children of other parts of the world [7] and a similar rise is seen in insulin resistance [8]. These two factors could be the reason for the high prevalence of obesity-related metabolic complications among Sri Lankan children [4]. Severity of childhood obesity scales up the magnitude of metabolic problems. Greater the severity of obesity, higher the risk of developing atherosclerosis, hypertension, dysglycaemia, type 2 diabetes, metabolic syndrome, fatty liver disease, and premature death [9]. Obese children also have lower self-esteem, poor social functioning, and internalising behaviour problems compared with their non‐overweight peers. Even quality of life could be poor and comparable to a child with malignancy [10].

Two main obesity-related abnormalities that have long-lasting consequences are cardiovascular diseases, and nonalcoholic fatty liver disease [11]. The Bogalusa heart study showed that children with severe obesity had a 2.7-fold greater risk of developing hypertension compared to their moderately obese counterparts [12]. Six cardiovascular risk factors were evaluated in this cohort of school children: low-density lipoprotein, triglycerides, high-density lipoprotein, cholesterol, fasting insulin, and systolic and diastolic blood pressure. This evaluation demonstrated that 39% of children with moderate obesity and 59% of children with severe obesity had at least two cardiovascular risk factors [12].

The spectrum of nonalcoholic fatty liver disease ranges from reversible hepatic macrovascular steatosis without inflammation to irreversible fibrosis and cirrhosis [13]. It is considered to be the commonest liver disease in children at present. It is documented that NAFLD prevalence varies from 3% among the general population of children to 80% in obese children [14]. Furthermore, in children, NAFLD is commonly seen among males during puberty and is associated with insulin resistance [15]. A Sri Lankan study estimated the presumed NASH to be about 18% among obese children [16]. Most of the children with NAFLD are asymptomatic or have nonspecific symptoms such as vague abdominal pain. The gold standard of diagnosis is liver biopsy, but it is invasive. In the absence of specific biochemical tests, elevated ALT/AST ratio >1 is suggestive of NASH [17]. Ultrasound scan (USS) is used to screen for NAFLD and has a predictive value of 84–94% [18]. A recent large prospective paediatric cohort showed a good correlation between steatosis score assessed by USS and the severity of steatosis on liver biopsy [19]. However, a recently developed transient elsastography (FibroScan®) and ultrasound eleastography using 2D shear wave elestography and point shear wave elastograpy can detect liver fibrosis and fatty liver noninvasively in both children and adults [20]. However, these equipments are not freely available in resource-poor settings and each scan is also costly. Further studies have shown that ultrasound scan used with standardized scoring in children does not differ from results obtained by FirbroScan® [21] Therefore, in the absence of a liver biopsy, a combination of steatosis detected by USS and an ALT/AST > 1 is a reasonable tool to diagnose NASH in a resource-poor setting.

Sex differences in obesity and related metabolic abnormalities have been highlighted in several studies [22]. The mechanism underlying the sex differences in cardiovascular risk factors (CVRF) is not clearly understood. Factors such as hormonal milieu influenced by fat content and gonadal maturity [23], X-chromosome dosage [24], genes of obesity and lipid metabolism largely drive the occurrence of childhood MetS [25].

As incidence of obesity is increasing and also insulin resistance and metabolic derangements are seen more frequently among the Sri Lankan population, this study was aimed to describe the metabolic derangements associated with obesity in a cohort of overweight and obese children in an urban setting of Sri Lanka.

## 2. Materials and Methods

### 2.1. Study Design and Setting

A cross-sectional study was conducted in the Negombo educational zone in the Gampaha district (Western province) of Sri Lanka.

### 2.2. Study Population

Overweight/obese (BMI for Age-SDS ≥+1SD, WHO 2007) [26] 7- to 17–year-old children were identified through a separate screening programme carried out in schools in the Negombo educational zone and were invited to participate [5]. Children having a secondary cause for overweight/obesity or suffering from long-term illness or on long-term medication were excluded.

### 2.3. Sample Size

Sample size was calculated to detect the expected prevalence of metabolic abnormalities in obese children. According to the findings of Wickramasinghe et al. [4], the prevalence of metabolic abnormalities among obese children was 19.2%. Using this estimated prevalence, an alpha error of 5% and a margin of error of 4%, the total sample required was calculated as 372. Adding a nonresponse rate of 10%, the final sample size required was determined as 409.

### 2.4. Data Collection Method

By appointment, study subjects were invited to the Diabetes Screening and Vocational Training Centre of the Lions Club of Negombo Host, Negombo. Informed written consent was obtained from the parents and assent was obtained from the children. After obtaining consent, participants were given an appointment to visit the data collection center.

After a 12-hour overnight fast, children visited the center, and height, weight, and waist circumference (WC) were measured by trained research assistants using a standardized protocol [27]. The FM was assessed by Bioelectrical impedance assay (BIA) using a platform type, eight electrode InBody 230® instrument (InBody®, Biospace, South Korea), which had been previously validated against locally developed BIA prediction equation [28]. Blood pressure was measured manually using a mercury sphygmomanometer in the seated position after 10–15 minutes of rest. If the reading was high, it was rechecked after 15 minutes of rest and the second reading was taken.

Blood was drawn to assess fasting blood glucose (FBG), serum total cholesterol, LDL-c, HDL-c, triglyceride, insulin, alanine transaminase (ALT), aspartate transaminase (AST), and high-sensitive C-reactive protein (Hs-CRP). Oral glucose tolerance test (OGTT) was performed after administering anhydrous glucose at the rate of 1.75 g/kg body weight to a maximum of 75 g and blood was drawn 2 hours later for random blood glucose (RBG) and serum insulin. Serum was separated immediately and stored at −20°C and analysis was conducted at the biochemical laboratory of the same center in batches. Blood was drawn after applying lignocane (Emla®) anesthetic cream. Insulin resistance was calculated using the homeostatic model (HOMA-IR = fasting blood sugar (mmol/L) × fasting insulin (microU/ml) / 22.5) [29].

An experienced radiologist assessed hepatic steatosis ultrasonically. Hepatic steatosis was categorized from Grade I to III [19]. NASH was diagnosed in the presence of hepatic steatosis and elevated ALT/AST ratio of ≥1.0.

Pubertal staging was assessed using visual charts [30]. Girls were shown a diagram with stages of breast and pubic hair development and were asked to match it with their own. Similarly, boys were shown diagrams of the development of different stages of external genitalia and pubic hair and were requested to match with their own. The size of the testis was measured by the examiner using a Prader orchidometer. If the subject or parents were not sure of the staging, with consent, the examiner assessed the pubertal stage for verification. Female and male investigators carried out measurements and examinations on the respective gender groups. In most of the cases, the investigators verified the results on the request of the parents.

### 2.5. Statistical Analysis

Standard descriptive methods, including frequencies, percentages, means, and standard deviations, were used to describe the data. Independent samples *T* test and chi-square test were used to compare the parameters between girls and boys. Hepatic factors associated with metabolic syndrome were analysed using binary logistic regression. Mean metabolic parameters in different BMI categories were compared using one-way ANOVA test.

### 2.6. Definition of Metabolic Syndrome (MetS)

IDF consensus definition to diagnose MetS in children was modified to suit all ages [28, 31]. Metabolic derangements were identified as: WC/Age-SDS ≥+2 [32]; abnormal glucose homeostasis, if FBG > 5.6 mmol/L (100 mg/dl) or 2-hour OGTT > 7.8 mmol/L (140 mg/dl); HDL < 1.03 mmol/L (<40 mg/dl); triglyceride ≥ 1.7 mmol/L (≥150 mg/dl) [33], and SBP-SDS ≥+2SD or DBP-SDS ≥+2SD [34]. MetS was diagnosed in the presence of a high WC, with 2/4 other criteria being elevated.

### 2.7. Cutoff Values for Metabolic Derangements

Fasting insulin >83 pmol/L (12 microIU/ml) [35] and 2-hour insulin >520 pmol/L (75 microIU/ml) [36] or HOMA-IR > 2.5 [37], ALT > 40 IU/L, AST > 40 IU/L, ALT/AST ratio > 1.0, and Hs-CRP >3 mg/L.

Data were collected from July to November 2014. Ethics approval was obtained from the Ethics Review Committee of the Faculty of Medicine, University of Colombo (EC-13-143).

## 3. Results

Data of 403 (boys 210) overweight and obese children were analysed. The cohort was desegregated based on age to 7–10 years (*n* = 112, boys 61) and 11–17 years (*n* = 291, boys 149).  Table 1 shows the distribution of the anthropometric, body composition, and metabolic parameters for each sex in each age category. Although the mean BMI SD score was not very high above the cutoff value of +2SD, the mean percentage fat mass was very high and girls had a significantly higher fat content than their male counterparts in each age group. Most of the parameters did not show a sex difference in the younger age group. In the older age group, girls were significantly shorter and had larger WC SD scores than boys. The mean total cholesterol levels in all four groups were above normal level but with minimum difference between groups. However, the mean HDL-c was very high in all four groups. Mean ALT was higher than the mean AST levels and both were significantly higher in boys than girls in the older age group. The mean fasting and random insulin levels were very high with high HOMA-IR levels, with no significant difference between the sexes.

Table 2 shows the distribution of abnormal anthropometric, body composition, and metabolic parameters of the population. Based on WHO BMI for Age SD score cutoff, 16.4% of the study population was overweight (BMI SD + 1 to +2SD), 83.6% was obese (BMI SD >+2SD), and 11.6% of the study population was severely obese (BMI SD >+3SD). Ninety-four percent had a WC above +2SD and 95% had a WHtR > 0.5. Elevated diastolic BP was seen in 16.8% of the population, but only 2.5% had elevated systolic BP. Insulin resistance was seen in 47% and seven children had diabetes mellitus with six of them in the older age group. Elevated 2-hour random insulin was seen in 35% of children denoting that there are many who require large amounts of insulin to maintain normoglycaemia. More than a third of children under 10 years of age and half of the older children showed insulin resistance.

Although a higher number of children had elevated non-HDL lipids, only 8% showed reduced HDL-c levels. More children had elevated ALT levels than elevated AST levels, and the elevated ALT/AST ratio was seen in 58% of the study population. About one-third of the study population had fatty livers with majority being in grade I fatty liver state. Elevated ALT and fatty liver were significantly more common in boys than in girls in the older age group.

Table 3 shows the distribution of different metabolic states according to age and sex. Dyslipidaemia was the commonest metabolic derangement (76.6%) and dysglycaemia was seen in 20.8% while 16.8% had hypertension. MetS was seen in about 19.8%% of this obese study group. Although MetS was seen only in about a fifth of this overweight/obese childhood and adolescent population, more than four-fifth of children had at least one abnormal metabolic component. At least one abnormal metabolic problem was seen in 84% of the population and 66.5% had at least two abnormal metabolic parameters. Therefore, only 33.5% could be considered metabolically normal obese and those having two or more abnormal metabolic parameters, were considered as unhealthy.

Table 4 shows the association between MetS and markers of nonalcoholic fatty liver disease (NAFLD). Although ALT and AST in isolation did not show any association with MetS, elevated ALT/AST ratio (OR = 2.12, *P*=0.005) and hepatic steatosis (OR = 1.84, *P*=0.015) were clearly associated with MetS. After adjusting for age and sex, the presence of elevated ALT/AST ratio (OR = 2.17 CI, 1.25–3.77, *P*=0.006) and having fatty liver disease (OR = 1.77, CI, 1.06–2.97, *P*=0.029) continued to be significantly associated with MetS.

Table 5 shows the mean distribution of the metabolic parameters across different degrees of overweight. Liver enzymes and measures of dysglycaemia showed significant increase with worsening degrees of overweight in both groups. However, the lipids did not show any significant relationship with the degree of overweight.

## 4. Discussion

Obesity, in a majority, is an illness caused by energy imbalance, where excess energy intake and low energy expenditure leads to energy surplus, which is deposited as fat for later use. When deposition occurs beyond a critical point, it leads to obesity. The incidence of obesity is on the rise and related metabolic abnormalities are increasingly seen among younger age groups. Furthermore, these abnormal metabolic markers in childhood are shown to have extended into adulthood and predict atherosclerosis and vascular dysfunction in later life [38]. One of the main complications of obesity is the clustering of cardiovascular risk factors primarily mediated through insulin resistance, which was initially described by Gerald Reaven at the 1988 Bantin lecture. It was initially described using different nomenclatures, and finally, the term MetS was chosen [39].

The overall MetS prevalence was 19.6% in this cohort and the distribution did not vary very much depending on the age. A previous study showed the prevalence of MetS to be 22.1% among obese children in the Colombo district where obesity was diagnosed based on IOTF cutoffs [4]. Eighty-five percent of the current sample had at least one metabolic abnormality. However, the prevalence of metabolic abnormalities depended on the study population and the BMI cutoff used to define them. A previous study showed all obese children to be having at least one metabolic derangement when obesity diagnosis was based on IOTF cutoff, and 43% having at least one abnormality when Sri Lankan BMI cutoffs were used [4]. The poor sensitivity of some of the international BMI cutoffs in diagnosing obesity would result in under diagnosing obesity, thus missing early stages of development of metabolic derangements [28].

A Finnish study on 2- to 17-year-old obese children showed the prevalence of hypertension and dysglycaemia to be 16.3% and 7.2%, respectively. However, insulin resistance measured by HOMA-IR was 43.8% [40]. Hypertension (16.8%) and insulin resistance (47%) in our study were similar; however, dysglcaemia (20.8%) in our cohort was very high. The same study saw dyslipidaemia in 35% but in our population, it was as high at 76%. These could be due to a reflection of probably a genetic predisposition to both insulin resistance and dyslipidaemia in this South Asian population where for a given BMI value, the body fat content is higher compared to the White Caucasian populations, and most of the body fat in Asians is deposited in the abdomen [7, 41].

Two to six-year-old children in the same Finish study showed a high WC in 85.1%, dyslipidaemia in 20.6%, hypertension in 5.9%, and IFG or IGT in 3.8%. A fifth of the population (19.8%) had abnormal HOMA showing a high amount of insulin resistance at such a young age [40]. Although we did not study such a younger age group, the younger population in our study, 7- to 10-year-old children, showed a very high prevalence of metabolic abnormalities compared to this younger Finish cohort, which could expect to match or even surpass if we would have studied a similar younger population.

A Polish study involving 6-, 10-, and 14-year-old overweight and obese children found that 38.2% of girls and 40.5% of boys with overweight and obesity were having at least one lipid abnormality. The most common lipid disorders that were observed were decreased HDL-c (20.6% of the girls and 23.8% of the boys) and elevated LDL-c (15.3% of the girls and 14.3% of the boys) [42]. Abnormal triglyceride was observed in a minority of children (6.0% of the girls and 8.8% of the boys). Those with dyslipidaemia were significantly older, had higher BMI, WC, and body fat than those without dyslipidaemia [42]. However, in our population, low HDL-c levels were less than ten percent but other lipid abnormalities were seen in more than a third to half of the study population.

Prevalence of MetS ranged from 15% to 25% in the different age and sex groups in the current study. A study in France involving 7- to 15-year-olds showed MetS to be 18.6% in children below 10 years of age and 14.5% among 10- to 15-year-old children according to the National Cholesterol Education Program (NCEP) Adult Treatment Panel III [43]. Based on IDF consensus definition, in 10- to 15-year-old children, the prevalence of MetS was 8.9%. Although there is still no consensus on the diagnostic criteria of MetS in children, it is quite evident that children with MetS pose a higher risk of developing MetS as adults. The Princeton prevalence and follow-up study has showed that childhood MetS has an odds ratio of 9.4 to develop MetS and 11.2 to develop diabetes mellitus as an adult [44]. For adults with MetS, the hazard ratio to develop coronary heart disease mortality was 2.87 and it was 5.02 for diabetes mellitus mortality [45].

Components of MetS such as obesity, insulin resistance, hypertension, and dyslipidaemia are seen to be clustered in black and white children and adults in Bogalusa, USA [38]. These results suggest that the MetS components coexist, not only in terms of absolute levels in childhood and adulthood but also in terms of rates of change from childhood to adulthood. This has shown the importance of obesity in the development of MetS in all ages, thus showing that maintaining a proper BMI from early life is of paramount importance in the prevention of obesity and related complications [38].

Data in our study quite clearly show that cardio metabolic risks and MetS are present in children as young as 7–10 years of age. Since evidence points out that obesity and metabolic complications persist into adulthood, early diagnosis and treatment of MetS and other metabolic abnormalities during childhood is important. Although there is lack of consensus on the definition of MetS in children, the American Diabetes Association and American Heart Association highlight that extra vigilance in detection of insulin resistance, glucose intolerance, and type 2 diabetes early in the course of development would lead to effective early interventions, thus preventing progression into complications [46]. Current knowledge shows that better glycaemic control would lead to better long-term microvascular and macrovascular outcomes, which are the prime factors that determine long-term outcomes of diabetes mellitus. Therefore, the best preventive approach for future cardiovascular disease is early recognition and aggressive intervention. Without this, it is likely that this patient population is destined to develop cardiovascular complications at a younger age in epidemic proportions [46].

The IDF consensus definition did not recommend to entertain a diagnosis of metabolic syndrome in children younger than 10 years of age due to lack of evidence [31]. However, it needs to be highlighted that this definition is now more than 14 years old and a dearth of evidence has been generated thereafter [4, 11, 40, 42]. The South Asian region where insulin resistance and CVRF are highly prevalent has produced lot of data on the magnitude of the problem of childhood obesity and related metabolic derangements [8, 37]. Therefore, in order to facilitate awareness and early intervention, it is important to make the diagnosis of MetS early in life.

Sex and pubertal status influences the occurrence of CVRF in children with obesity [40]. Different CVRFs predominate in both the genders differently with more insulin resistance seen in females and hypertension, abdominal obesity, and dysplipdaemia in males, and further risk factors were seen in prepubertal age as well [40]. However, in our study, no significant sex difference was noted for metabolic derangements except for the fat content where females had a higher amount of body fat compared to their male counterparts, especially abdominal fat in the older age group, denoted by the high WC, SD, and WHtR. Pubertal status did not show significant influence on the occurrence of metabolic abnormalities with prepubertal children also having high prevalence of metabolic derangements and MetS in our study (data not shown). The mechanism underlying the sex differences in CVRF is not clearly understood but a multitude of factors could be affecting where genetics could be playing a major role. In our data, we do not see a clear relationship of lipid abnormalities with the degree of overweight as well as with sex, but insulin resistance and related metabolic abnormalities including liver damage increased with the worsening degree of overweight as well as with increasing age. This probably could shed some light on the fact that genetics of obesity (or insulin resistance) and lipid metabolism could act in harmony to develop metabolic derangements in children [25]. Therefore, probably in this South Asian population, a multitude of factors such as hormone milieu driven by fat content, genetics determining regional body composition, obesity, and lipid metabolism could be playing a role in developing metabolic abnormalities of a higher magnitude at a younger age.

The purpose of screening for NAFLD in children and adolescents is to prevent irreversible, end-stage liver disease. The exponential rise in childhood overweight/obesity has made NAFLD one of the most important causes of chronic liver disease [47] However, NAFLD in children is still underdiagnosed mainly due to poor awareness, recognition, and due to the absence of a proper validated diagnostic tool [48, 49]. White Caucasians and Asian children have high prevalence compared to African American children, and NAFLD is also more prevalent in male children than female children [48, 50, 51]. Based on a meta-analysis of evidence from studies conducted in paediatric obesity clinics, the prevalence of NAFLD among children and adolescents was 34.2% [52]. In our population of overweight and obese children, prevalence of NAFLD was 32.5% and that of NASH was 26.5%, which are similarly high. With age, children are more likely to have complications of NAFLD and there is an urgent need to develop robust screening tools for early detection in high-risk groups [49].

## 5. Conclusions

This study clearly shows that cardiovascular metabolic risk, MetS, NAFLD and NASH are prevalent among obese Sri Lankan children starting from a very young age. This warrants active screening of the child population for obesity and assessing for both cardiovascular and hepatic metabolic complications actively in order to prevent the progression of obesity-related metabolic derangements into adulthood and to curb the ever-increasing rates of NCD in the world. Furthermore, reaching a consensus on diagnostic techniques for obesity and MetS and creating awareness about these conditions among the medical fraternity as well as the general public is of paramount importance and urgency.

## Figures and Tables

**Table 1:** Mean distribution of anthropometric, body composition, and metabolic parameters according to sex and age group.

Characteristics	7–10 years			11–17 years		
	Males (*n* = 61)	Females (*n* = 51)	*P* value^*∗*^	Males (*n* = 149)	Females (*n* = 142)	*P* value^*∗*^
	Mean (SD)	Mean (SD)		Mean (SD)	Mean (SD)	
Height SD score	0.67 (0.89)	0.54 (0.97)	0.45	0.13 (1.07)	−0.24 (0.89)	**0.002**
BMI SD score	2.75 (0.61)	2.51 (0.5)	**0.028**	2.37 (0.48)	2.31 (0.38)	0.254
WC SD score	2.89 (0.55)	2.88 (0.39)	0.91	2.61 (0.49)	3.13 (0.5)	**<0.001**
Waist/height ratio	0.57 (0.04)	0.56 (0.04)	0.30	0.58 (0.05)	0.57 (0.04)	**0.032**
Percentage FM	40.8 (4.6)	44.0 (4.11)	**<0.001**	39.4 (5.8)	44.6 (4.51)	**<0.001**
Systolic BP SD score	−0.75 (1.16)	−0.94 (0.79)	0.34	−0.74 (1.07)	−0.67 (1.03)	0.59
Diastolic BP SD score	0.83 (1.09)	0.69 (1.03)	0.49	1.11 (1.01)	1.24 (0.79)	0.23
FBS (mmol/L)	4.83 (0.49)	4.82 (0.51)	0.94	4.93 (0.51)	4.92 (0.69)	0.89
Fasting insulin (pmol/L)	98.62 (157.65)	138.21 (291.0)	0.38	128.48 (204.9)	115.98 (164.6)	0.58
2-hour RBS (mmol/L)	5.96 (0.85)	6.20 (1.32)	0.24	6.49 (1.32)	6.64 (1.86)	0.42
2-hour insulin (pmol/L)	368.09 (367.39)	434.76 (322.25)	0.35	586.16 (474.3)	620.88 (450.7)	0.55
HOMA-IR	3.13 (5.5)	4.43 (10.2)	0.41	4.04 (6.1)	3.64 (4.9)	0.57
Total cholesterol (mmol/L)	5.62 (0.89)	5.47 (1.03)	0.41	5.41 (1.12)	5.56 (1.18)	0.27
Triglycerides (mmol/L)	1.65 (0.59)	1.67 (0.65)	0.81	2.85 (0.58)	1.59 (0.56)	0.061
LDL cholesterol (mmol/L)	3.43 (0.73)	3.27 (0.88)	0.3	3.30 (0.97)	3.43 (0.95)	0.25
HDL cholesterol (mmol/L)	1.43 (0.30)	1.43 (0.32)	0.95	1.33 (0.30)	1.41 (0.36)	**0.043**
Hs-CRP (mg/L)	1.02 (0.75)	1.39 (0.94)	**0.022**	1.12 (0.88)	1.02 (0.78)	0.28
ALT (IU/L)	34.6 (36.9)	27.6 (17.1)	0.22	33.2 (22.0)	23.6 (19.4)	**<0.001**
AST (IU/L)	29.5 (18.7)	25.9 (10.8)	0.23	26.9 (11.5)	21.1 (9.4)	**<0.001**

**Table 2:** Distribution of abnormal anthropometric, body composition, and metabolic parameters according to sex and age group.

Characteristics		7–10 years			11–17 years		
		Males (*n* = 61)	Females (*n* = 51)	*P* value^*∗*^	Males (*n* = 149)	Females (*n* = 142)	*P* value^*∗*^
		*N* (%)	*N* (%)		*N* (%)	*N* (%)	
Height SD score > 2		6 (9.8)	2 (3.9)	0.23	9 (6.0)	2 (1.4)	0.038
Stunting (height SDS < −2)		0 (0.0)	0 (0.0)	—	3 (2.0)	3 (2.1)	0.95
BMI SD score	1-2	10 (16.4)	8 (15.7)	0.175	24 (16.1)	24 (16.9)	0.31
	2-3	31 (50.8)	34 (66.7)		112 (75.2)	112 (79.8)	
	>3	20 (32.8)	9 (17.6)		13 (8.7)	6 (4.2)	
WC SD score > 2		56 (91.8)	50 (98.0)	0.30	135 (90.6)	138 (97.2)	0.02
Waist-height ratio > 0.5		57 (93.4)	49 (96.1)	0.54	141 (94.6)	136 (95.8)	0.65
High percentage FM^a^		61 (100.0)	51 (100.0)	—	143 (96.0)	142 (100.0)	0.03^#^
Systolic BP SD score > 2		2 (3.3)	1 (2.0)	0.67	3 (2.0)	4 (2.8)	0.66
Diastolic BP SD score > 2		12 (19.7)	6 (11.8)	0.26	26 (17.4)	24 (16.9)	0.9
Impaired FBG^b^		6 (9.8)	7 (13.7)	0.52	16 (10.7)	18 (12.7)	0.87^##^
Impaired 2-hour RBG^j^		2 (3.3)	3 (5.9)	0.43^#^	18 (12.1)	21 (14.8)	0.69^##^
Diabetes mellitus^*∗∗*^		0 (0.0)	1 (2.0)	0.92	3 (2.0)	3 (2.1)	1.0
Fasting insulin > 83 pmol/L		22 (38.6)	16 (33.3)	0.58	70 (50.7)	70 (56.5)	0.35
2-hour Insulin > 520 pmol/L		13 (24.1)	18 (40.0)	0.089	49 (37.7)	61 (49.2)	0.064
High HOMA-IR^k^		26 (42.6)	17 (33.3)	0.31	73 (49.0)	73 (51.4)	0.68
High total cholesterol^c^		44 (72.1)	28 (54.9)	0.058	81 (54.4)	93 (65.5)	0.053
Elevated triglycerides^d^		25 (41.0)	18 (35.3)	0.54	67 (45.0)	45 (31.7)	**0.02**
Elevated LDL^e^		33 (54.1)	22 (43.1)	0.25	64 (43.0)	74 (52.1)	0.12
Low HDL^f^		5 (8.2)	2 (3.9)	0.59	17 (11.4)	9 (6.3)	0.13
Elevated Hs-CRP^g^		1 (1.6)	5 (9.8)	0.14	6 (4.0)	4 (2.8)	0.57
Elevated ALT^h^		12 (19.7)	6 (11.8)	0.25	32 (21.5)	15 (10.6)	**0.011**
Elevated AST^h^		7 (11.5)	5 (9.8)	0.78	14 (9.4)	6 (4.2)	0.81
High ALT/AST ratio^i^		34 (55.7)	27 (52.9)	0.85	101 (67.8)	72 (50.7)	0.003
Fatty liver, grade I		11 (18.0)	8 (15.7)	0.68	39 (26.2)	20 (14.1)	**0.001**
Fatty liver, grade II		6 (9.8)	3 (5.9)		22 (14.8)	14 (9.9)	
Fatty liver, grade III		0 (0.0)	0 (0.0)		7 (4.7)	1 (0.7)	

^a^Percentage of fat mass >28.6% in boys and >33.7% in girls (Wickramasinghe et al.). ^b^Fasting blood glucose 5.6–6.9 mmol/L. ^c^Total cholesterol > 5.2 mmol/L. ^d^Triglycerides > 1.7 mmol/L. ^e^LDL cholesterol > 3.4 mmol/L. ^f^HDL cholesterol < 1.03 mmol/L. ^g^High-sensitivity C-reactive protein >3 mg/L. ^h^Alanine transaminase (ALT) and aspartate transaminase (AST) > 40 IU/L. ^i^ALT/AST > 1. ^j^2-hour random blood glucose 140–200 mg/dL. ^k^HOMA-IR > 2.5. ^*∗∗*^Fasting blood glucose > 6.9 mmol/L or 2-hour random blood glucose > 11.1 mmol/L. ^#^Using Fisher's exact test. ^##^Significance tested amalgamating IFG and DM categories. In the 7–10 years age group, 1 girl and 2 boys had both systolic and diastolic BP elevated. In the 11–17 years age group, 4 girls and 2 boys had both systolic and diastolic BP elevated. In the 11–17 years age group, 6 girls and 5 boys had both impaired FBG and impaired 2-hour RBG. In the 11–17 years age group, 1 girl had both FPG and 2-hour RBG elevated to the level of diabetes mellitus. In the 5–10 years age group, 7 girls and 6 boys had both fasting and 2-hour insulin elevated. In the 11–17 years age group, 37 girls and 35 boys had both fasting and 2-hour insulin elevated. BMI: body mass index; WC: waist circumference; BP: blood pressure; FBS: fasting blood sugar; RBS: random blood sugar; HOMA-IR: homeostatic model assessment of insulin resistance; Hs-CRP: high-sensitivity C-reactive protein; ALT: alanine transaminase; AST: aspartate transaminase.

**Table 3:** Prevalence of metabolic abnormalities according to sex and age group.

Metabolic abnormality		7–10 years			11–17 years		
		Males (*n* = 61)	Females (*n* = 51)	*P* value^*∗*^	Males (*n* = 149)	Females (*n* = 142)	*P* value^*∗*^
		*N* (%)	*N* (%)		*N* (%)	*N* (%)	
Dysglycaemia^a^		8 (13.1)	10 (19.6)	0.35	30 (20.1)	35 (24.6)	0.35
Dyslipidaemia^b^		53 (86.9)	32 (62.7)	**0.003**	119 (79.9)	104 (73.2)	0.18
Elevated BP^c^		12 (19.7)	6 (11.8)	0.26	26 (17.4)	24 (16.9)	0.90
Metabolic syndrome^d^	Total	10 (16.4)	8 (15.7)	0.91	38 (25.5)	24 (16.9)	0.073
	Overweight	1 (10.0)	2 (25.0)	0.83	6 (25.0)	2 (8.3)	0.24
	Obese	9 (17.6)	6 (14.0)	0.63	32 (25.6)	22 (18.6)	0.19

No. of metabolic abnormalities^e^							
None		7 (11.5)	13 (25.5)	0.13^#^	22 (14.8)	23 (16.2)	0.25^#^
1		7 (11.5)	7 (13.7)		27 (18.1)	30 (21.1)	
2		28 (45.9)	14 (27.5)		54 (36.2)	36 (25.4)	
3		13 (21.3)	12 (23.5)		29 (19.5)	33 (23.2)	
4		4 (6.6)	5 (9.8)		16 (10.7)	17 (12.0)	
5		2 (3.3)	0 (0.0)		1 (0.7)	3 (2.1	

^a^Fasting blood glucose > 5.6 mg/dL or 2-hour random blood glucose > 7.8 mg/dL. ^b^Total cholesterol > 5.2 mmol/L or LDL cholesterol > 3.4 mmol/L or triglycerides > 1.7 mmol/L or HDL cholesterol < 1.03 mmol/L. ^c^Systolic blood pressure and/or diastolic blood pressure >2 SD scores for age and sex. ^d^Waist circumference >2 SD scores plus 2 of the following criteria: systolic or diastolic BP elevated above 2 SD scores; serum triglycerides > 1.7 mmol/L; serum HDL cholesterol < 1.03 mmol/L; dysglycaemia (FBG > 5.6 mmol/L or 2-hour RBG > 7.8 mmol/L). ^e^Number of metabolic abnormalities out of elevated BP (systolic or diastolic BP > 2 SDS), dysglycaemia (FBG > 5.6 mmol/L or 2-hour RBG > 7.8 mmol/L), elevated total cholesterol (>5.2 mmol/L), elevated LDL (>13.4 mmol/L), elevated triglycerides (>1.7 mmol/L), low HDL (<1.03 mmol/L), and elevated Hs-CRP (>3 mg/L). ^*∗*^*P* value calculated using chi-square test. ^#^Statistical significance was calculated after amalgamating 3, 4, 5, and 6 metabolic abnormalities in order to account for small values in cells.

**Table 4:** Association between the presence of metabolic syndrome and liver abnormalities (*n* = 403).

Liver abnormalities		Metabolic syndrome		OR (95% CI) significance^*∗*^	Adjusted OR (95% CI) significance^*∗∗*^
		Absent (*n* = 323) *N* (%)	Present (*n* = 80) *N* (%)		
ALT	Normal	275 (81.6)	62 (18.3)	1.71 (0.93–3.14); *P* = 0.084	1.73 (0.94–3.21); *P* = 0.079
	High	47 (72.3)	18 (27.7)		
AST	Normal	299 (80.8)	71 (19.1)	1.65 (0.73–3.7); *P* = 0.22	2.03 (0.9–4.57); *P* = 0.088
	High	23 (71.9)	9 (28.1)		
ALT/AST	Normal	147 (87.5)	21 (12.5)	**2.12 (1.25–3.61);** **P** ** = 0.005**	**2.17 (1.25–3.77);** **P** ** = 0.006**
	High	175 (74.8)	59 (25.2)		
Fatty liver	Absent	226 (83.7)	44 (16.3)	**1.84 (1.12–3.04);** **P** ** = 0.015**	**1.77 (1.06–2.97);** **P** ** = 0.029**
	Present	96 (73.3)	35 (26.7)		
NASH^a^	Absent	243 (82.9)	50 (17.1)	**1.73 (1.02–2.9)**; **P = 0.039**	1.15 (1.02–2.72); *P* = 0.093
	Present	78 (72.9)	29 (27.1)		

AST: aspartate transaminase; ALT: alanine transaminase. ^*∗*^Using chi-square test. ^a^NASH: nonalcoholic steatohepatitis is diagnosed in the presence of the fatty liver with elevated ALT/AST. ^*∗∗*^Using binary logistic regression with the presence of metabolic syndrome as the dependent variable and adjusting for age and sex. Data missing in *n* = 1 for AST and ALT, *n* = 2 for the fatty liver, and *n* = 3 for NASH.

**Table 5:** Patterns of metabolic parameters according to the BMI category.

Metabolic parameters	BMI SDS 1-2 (*n* = 66)	BMI SDS 2-3 (*n* = 289)	BMI SDS > 3 (*n* = 48)	Significance^*∗*^	Groups with significant difference
	Mean (SD)	Mean (SD)	Mean (SD)		
Females					

FBG (mmol/L)	4.81 (0.52)	4.92 (0.68)	4.76 (0.59)	0.49	—
Fasting insulin (pmol/L)	97.23 (77.09)	126.40 (229.19)	90.28 (65.28)	0.70	—
2-hour RBS (mmol/L)	6.05 (1.05)	6.60 (1.83)	6.27 (1.35)	0.23	—
2-hour insulin (pmol/L)	476.43 (295.86)	587.55 (437.54)	517.40 (329.89)	0.41	—
HOMA-IR	3.08 (2.5)	4.2 (7.5)	2.73 (2.1)	0.69	—
Total cholesterol (mmol/L)	5.50 (1.29)	5.52 (1.12)	5.80 (1.15)	0.65	—
Triglycerides (mmol/L)	1.64 (0.73)	1.64 (0.56)	1.34 (0.37)	0.15	—
LDL cholesterol (mmol/L)	3.36 (0.97)	3.36 (0.93)	3.72 (0.85)	0.35	—
HDL cholesterol (mmol/L)	1.39 (0.31)	1.41 (0.36)	1.47 (0.37)	0.78	—
Hs-CRP (mg/L)	0.92 (0.64)	1.1 (0.82)	1.5 (1.03)	0.064	—
ALT (IU/L)	19.5 (10.6)	24.3 (19.3)	37.5 (23.6)	**0.009**	1-2 and >3, 2-3 and >3
AST (IU/L)	19.5 (6.2)	22.2 (10.0)	28.6 (13.8)	**0.014**	1-2 and >3
ALT/AST ratio	0.98 (0.27)	1.05 (0.31)	1.25 (0.36)	**0.023**	1-2 and >3, 2-3 and >3
Percentage fat mass	40.3 (4.01)	44.83 (3.8)	49.85 (3.21)	**<0.001**	All 3 from each other

Males					

FBG (nmol/L)	4.86 (0.51)	4.92 (0.53)	4.91 (0.42)	0.82	—
Fasting insulin (pmol/L)	81.95 (71.53)	118.06 (182.65)	165.29 (293.77)	0.22	—
2-hour RBS (nmol/L)	5.84 (0.77)	6.40 (1.28)	6.65 (1.27)	**0.021**	1-2 and >3
2-hour insulin (pmol/L)	379.89 (377.11)	515.32 (432.67)	697.97 (563.24)	**0.023**	1-2 and >3
HOMA-IR	2.55 (2.1)	3.68 (5.3)	5.39 (9.9)	0.16	—
Total cholesterol (mmol/L)	5.57 (1.23)	5.43 (1.03)	5.47 (1.06)	0.73	—
Triglycerides (mmol/L)	1.73 (0.68)	1.69 (0.55)	1.71 (0.61)	0.94	—
LDL cholesterol (mmol/L)	3.29 (1.05)	3.33 (0.88)	3.40 (0.92)	0.89	—
HDL cholesterol (mmol/L)	1.48 (0.37)	1.33 (0.28)	1.35 (0.24)	**0.024**	1-2 and 2-3
Hs-CRP (mg/L)	0.83 (0.72)	1.1 (0.86)	1.4 (0.82)	**0.022**	1-2 and >3
ALT (IU/L)	25.7 (19.3)	33.2 (21.6)	43.1 (47.6)	**0.034**	1-2 and >3
AST (IU/L)	26.5 (13.6)	27.5 (11.7)	29.3 (22.1)	0.71	—
ALT/AST ratio	0.95 (0.32)	1.18 (0.38)	1.40 (0.54)	**<0.001**	All 3 from each other
Percentage fat mass	34.45 (5.06)	39.78 (4.56)	45.55 (4.09)	**<0.001**	All 3 from each other

^*∗*^Statistical significance was assessed using one-way ANOVA test, and the pairwise comparisons were made using the Bonferroni method.

## Data Availability

The data used to support the findings of this study are available from the corresponding author upon request.
